# Supplementary figures and images for: Towards mouse genetic-specific RNA-sequencing read mapping
Source: PLoS Comput Biol. 2022 Sep 26;18(9):e1010552. doi: 10.1371/journal.pcbi.1010552 (PMC9536569; doi:10.1371/journal.pcbi.1010552)

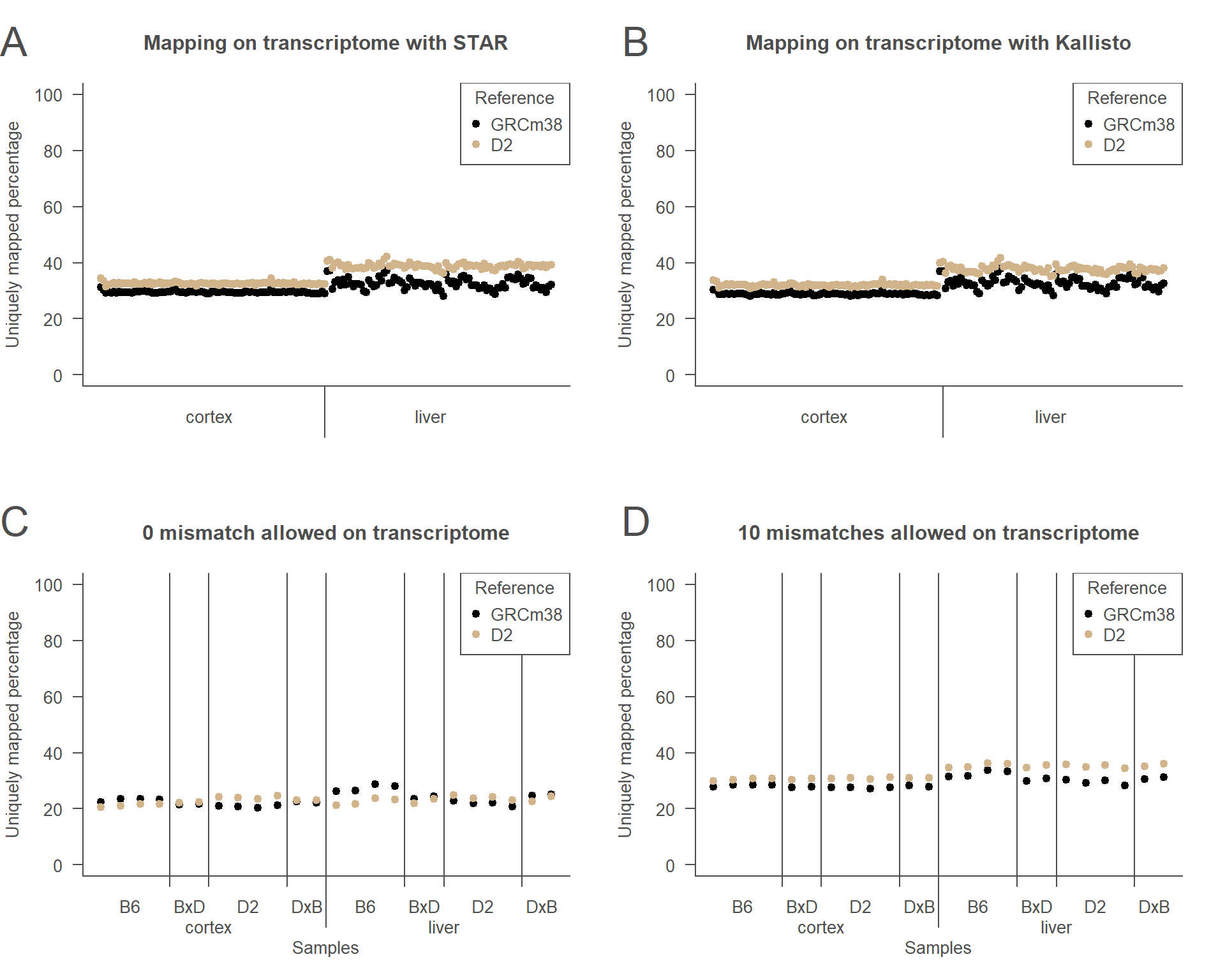

Supplement: S1 Fig — A. Mappability of all samples on 2 parental assembly transcriptomes using STAR permissive mapping setting. B. Pseudo-mappability of all samples on 2 parental assembly transcriptomes using Kallisto. C. Mappability of parental and F1 samples on 2 parental assembly transcriptomes using STAR restrictive mapping setting. D. Mappability of parental and F1 samples on 2 parental assembly transcriptomes using STAR restrictive mapping setting, but up to 10 mismatches. (TIFF) [file pcbi.1010552.s004.tiff]

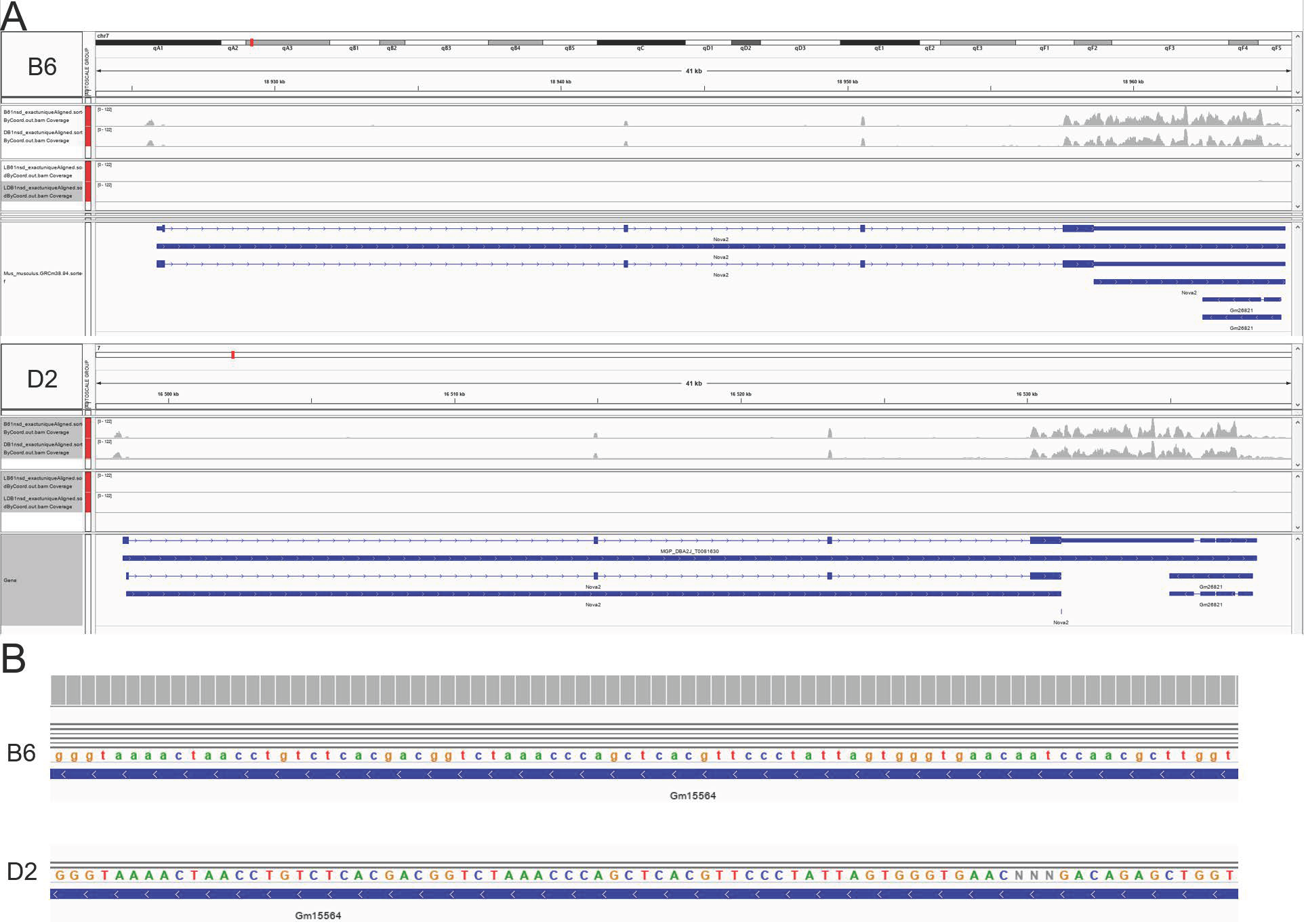

Supplement: S2 Fig — A. Nova2 genomic region in Integrative Genomics Viewer (IGV https://software.broadinstitute.org/software/igv/), as a transcriptome annotation artefact. The coverage is very similar between GRCm38 and D2 assemblies, but the annotation differs, which causes the reads to be counted differently. B. Gm15564 genomic sequence in IGV (https://software.broadinstitute.org/software/igv/), as an artefact due to difference in completeness of genome assembly. Many reads map to this region on GRCm38 assembly, but not on D2. It appears that in this region of the D2 assembly there are three unknown nucleotides (with label “N”), which supports the interpretation that it is probably due a difference in assembly quality, and not to a genomic variant. (TIFF) [file pcbi.1010552.s005.tiff]

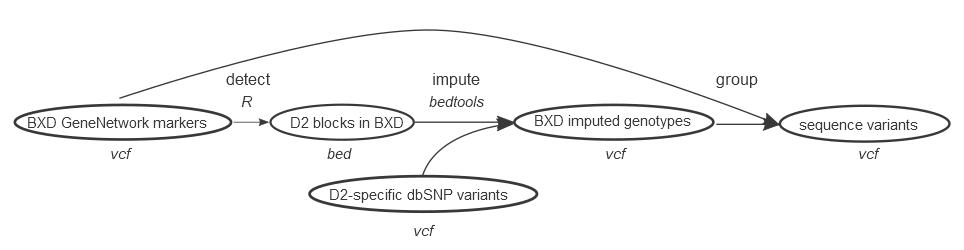

Supplement: S3 Fig — (TIFF) [file pcbi.1010552.s006.tiff]

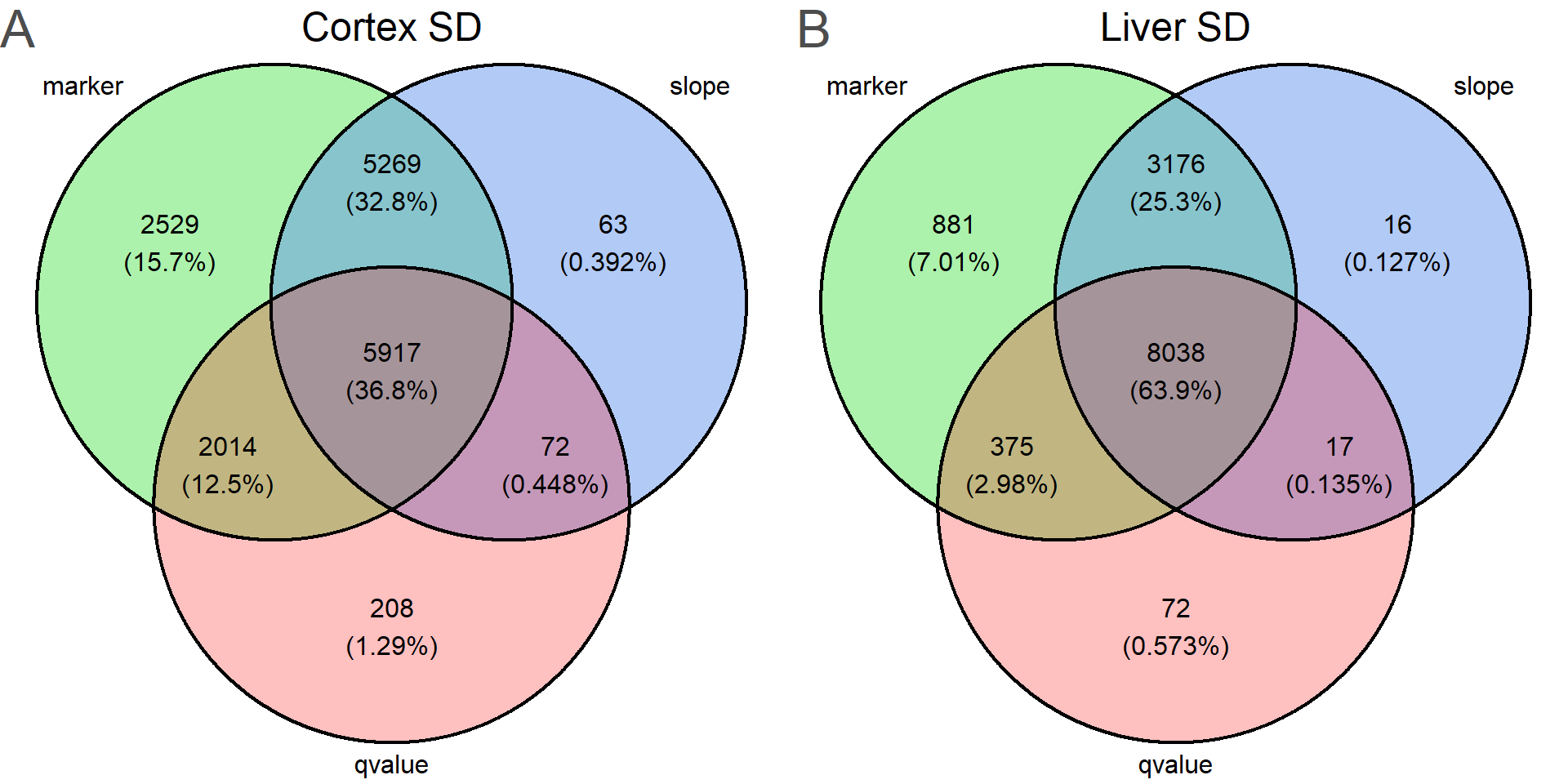

Supplement: S4 Fig — A. For all expressed genes, the best local genetic marker to explain gene expression is selected. The Venn diagrams represent the overlap of this analysis between GRCm38 and BXD-specific references for the three criteria in cortex SD. The marker (in green) indicates changing the reference result in the same genetic marker associated with gene expression. The slope (in blue) is the direction and strength of allele-specific gene expression, it is considered to be overlapping between the references if it varies less than 5%. The qvalue (in pink) is the statistical significance of the marker to gene expression association, it is considered to be overlapping between the references if it varies less than 5%. B. Same than A but in the liver SD. (TIFF) [file pcbi.1010552.s007.tiff]
