# Supplementary material for: Towards mouse genetic-specific RNA-sequencing read mapping
Source: PLoS Comput Biol. 2022 Sep 26;18(9):e1010552. doi: 10.1371/journal.pcbi.1010552 (PMC9536569; doi:10.1371/journal.pcbi.1010552)
Supplement: Attachment — Submitted filename: BXDmapping_answersreviewerscomments.pdf [file pcbi.1010552.s008.pdf]

Reviewer's Responses to Questions

Comments to the Authors:
Please note here if the review is uploaded as an attachment.

Reviewer #1: In this manuscript Gobet et al examine the impact the choice of reference genome and existing genetic variation has on downstream analyses. It is an interesting study with potential implications for downstream analyses in model organisms. The code is available and the methods are well described. While interesting, I found the manuscript requires clearer descriptions of the experiment, more examples of types of errors, and further discussion about what the results mean for both inbred and outbred organisms.
We thank reviewer #1 for their detailed review and constructive criticism.

Major comments:

1) Experimental design: While I eventually was able to understood the design the manuscript would benefit from clearer text and a flowchart describing the analysis. Figure 1 B attempts this but the legend does not sufficiently detail the image. This should be improved and presented at the onset. Further the 'hybrid' strategy (BXD-specific references) is poorly described and I am unclear what, if any analyses, were preformed using this strategy.

We clarified the legend of Figure 1B as follows: "The 3 RNA-seq read mapping strategies used in this study. In the 'two parental assemblies' strategy (left), the reads of all samples are mapped to the classical mouse genome assembly (GRCm38 or mm10) and to the D2 assembly. The 'BXD-specific references' (middle) were made from GRCm38 and BXD-specific variants. There is one reference for each BXD line, and the reads of each sample are mapped to the corresponding reference. The 'two parental references' (right panel) is an intermediate strategy in which the D2-specific reference is built from GRCm38 assembly and D2-specific variants." (lines 127-134).

The BXD-specific references were used in the figures 3 (except panel A), 4 and 5. We apologize for the confusion, we clarified by explicitly using the words "BXD-specific references" in the figure legends corresponding to results using this strategy (lines 369, 372, 393, 395, 398, 436). We also modified the following sentence in the result for the mapping evaluation: "We used the BXD-specific references, and to test which values to use for the more critical mapping parameters of STAR we varied: i) the number of mismatches allowed, ii) the possibility to trim end of reads, iii) the possibility to splice reads, and iv) the use of known transcriptome annotation." (lines 421-424).

2) The authors conclude the modified B6 reference is superior relative to the two reference approach however this is potentially just reflecting the lower quality of the D2 reference as you later mention. On L454 you state "However, we showed that the gap in quality and completeness between the two parental assemblies is masking the genetic specificity". While this may be true, you do not demonstrate this in the paper. In order to make any such conclusions, detailed information on the quality of the two references is required.

Here is presented summary information on the quality of the two parental genome assemblies, now supplementary table 2 in the manuscript. For example, 11% of nucleotides are N in DBA/2J (D2) assembly versus 3% for GRCm38.

	GRCm38	DBA/2J v1
Nb of sequences	66	4104
Nb of sequences >1kb	66	4102
Nb of sequences >10kb	65	624
Nb of nucleotides (counting Ns)	2730871774	2606145858
Nb of nucleotides U	0	0
Nb of sequences with U nucleotides	0	0
Nb of IUPAC nucleotides	0	0
Nb of sequences with IUPAC nucleotides	0	0
Nb of Ns	78088274	296844310
Nb of internal N-regions (possibly links between contigs)	591	409259
Nb of long internal N-regions >10000<br>(This is problematic for Genemark)	167	2954
Nb of pure (only) N sequences	0	0
Nb of sequences that begin or end with Ns	42	0
GC-content (%)	40.5	37
GC-content not counting Ns(%)	41.7	41.8
Nb of sequences with lowercase nucleotides	65	3888
Nb of lowercase nucleotides	1216679489	834025268
N50	130694993	128213663
L50	9	9
N90	94987271	93736615
L90	18	18

Table S1 | Summary statistics of the mouse genome assemblies
Assembly summary statistics calculated with GAAS toolkit (https://github.com/NBISweden/GAAS).

The main point we wanted to make is not the difference in the quality of the two assemblies per se, but that this difference masks the genetic specificity. We therefore have rephrased the statement in the discussion as follows: “However, we observed that the technical difference in quality between the two parental assemblies prevents a fair comparison of the genetic difference.” (lines 483-484). Although it was admittedly naive to try with the D2 assembly knowing it was less complete, we thought it was easier to test and show this (Figure 1B “two parental assemblies” and Figure 2) than to say it will not work.

3) In Fig S2 you provide a few examples of read mismapping largely due to differences in the assembly and annotation. While useful, it would be better to try to broadly classify the types of issues encountered via a diagram or table. For example, are gene families or repeat elements regions prone to mismapping? Perhaps looking at the cases in Figure 4 that actually change the eQTL slope would be insightful.

The issues in the examples in Figure S2 were identified by visual inspection of the alignments. We now determined how many of the differentially mapped (DM) genes seem to fit into these 2 types of issues. For this we quantified the number of DM genes with “N” nucleotides in the GRCm38 or D2 assembly, and how many DM genes had a different number of genes annotated in the locus between the two assemblies. The results have been added to the ms as supplementary table 3.

Tissue	DM genes	DM genes with "N" in GRCm38 assembly	DM genes with "N" in D2 assembly	DM genes whose locus overlap with a different number of genes in the 2 assemblies
Cortex	5228	0	558	1152
Liver	2456	0	645	648

Table S2 | Broad classification of DM genes according to the two types of artefacts identified.

These two issues explain a fraction of all DM genes. We also looked at the biotypes to see whether the pseudogenes would be enriched in DM genes compared to all expressed genes. We found that with the GRCm38 annotation, DM genes were enriched for pseudogenes (1.9-fold for cortex, 2.8 for liver) compared to all expressed genes, but with the D2 annotation the DM genes are depleted (0.6 for cortex, 0.9 for liver) in pseudogenes compared to all expressed genes. In any case, the majority of DM genes are not pseudogenes.

Visual inspection of cases that change the slope at Figure 4 did not give a clear result: some of the variants did seem to influence read mapping while others did not. Moreover, it seemed that it is not so much the reads that go from unmapped to uniquely mapped (or vice versa) but rather changes involving multi-mapped reads. The difficulty is that we do not know the true location of each read because they are real samples.

4) Many factors are known to have a significant impact on read mappability including the choice of aligner and read length. More discussion is needed on such factors and what (if any) impact they would be expected to have on the results of this study.

In supplementary Figure 1, we compared the results of two conceptually very different mapping softwares: STAR (classic aligner, (Dobin et al., 2013)) and Kallisto (pseudo-alignment mapper, (Bray et al., 2016)). The results of the two programs of mappability (percentage of uniquely mapped reads) were very similar and both showed the D2 bias for all samples. Based on our data and on the literature (Sha et al., 2015), we therefore decided that the choice of mapping software was not an important factor.

The read length is not expected to have an important impact other that smaller reads may be harder to uniquely map. There is no reason why the decision to use a more specific reference would be influenced by read length.

We extended the discussion as follows (lines 448-452):
"A great number of factors are involved, going from experimental design (number of replicates, kit for library preparation, sequencing platform, read length) to softwares, functions, and settings used in the different analytical different steps (quality control, mapping, filtering, normalization). We are not being exhaustive about all these aspects, but nevertheless think our observations and considerations on the selected factors we did address are useful for the community."

5) What is the impact on mappability of SNVs compared to small indels? While SNVs are more numerous indels are more likely to affect mapping. What portion of mismapped reads are attributable to each variant class?
As we do not have the true mapping locus of each read, we cannot know for sure which reads are mismapped, but we can use uniquely mapped reads as proxy of correctly mapped reads. In addition, to

attribute a read to a specific variant is not straightforward because a read may overlap with multiple variants, and variants not in the same region as the true locus can have an influence on the read alignment by making other regions more or less similar. One thing we can do is to compare read mappability of references that were modified by integrating different types of variants. Below we have illustrated this by quantifying the percentage of uniquely mapped reads for our four D2 samples in NSD condition (2 replicates per tissue, cortex on top, liver on bottom) to see which type of D2-specific variants needs to be modified in the GRCm38 assembly to have the highest mappability. We always comparing relatively to the GRCm38 assembly without modifications, and there are three different versions of D2; i.e., modified with either:

1. D2-specific indels from dbSNP ("indels"),
2. D2-specific SNVs from dbSNP ("SNVs"),
3. D2-specific SNVs and indels from dbSNP ("indelsSNVs").

We can see that the gain from SNVs (4.1% on average) is higher than the gain from indels (0.5% on average). Notice that the biggest gain is when we use both SNVs and indels (5.1% on average).

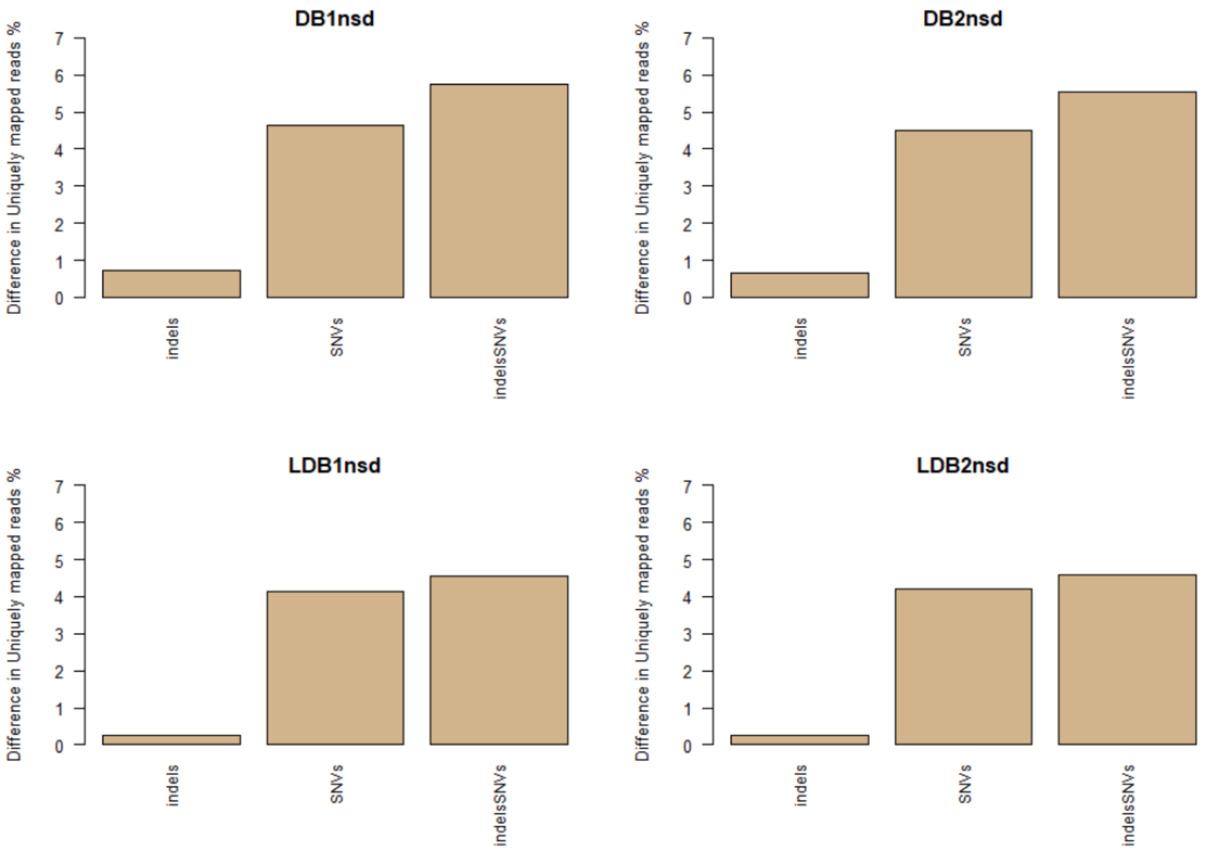

6) You recommend modifying mm10 with strain specific variants to achieve the best results. This is not a trivial undertaking however, does your code offer this functionality?

Our code is not needed to modify mm10 with strain specific variants. The tool that we used, vcf2diploid was released in 2014. It is not a task often performed, but it is not complex compared to the alignment and we think it is worth the trouble. The analyses steps would be as follows:

1) Download genome assembly sequence (fasta) and transcriptome (gtf)

2) Download variants (vcf)

3) Install aligner (e.g. STAR)

4) Install assembly modifier (e.g. vcf2diploid)

5) Generate variant-specific reference

6) Generate reference index for reference

7) Align reads

(underlined steps are the ones we suggest to add to the typical alignment steps)

We modified the method section to clarify that the modifications that we made are not required to run the tool that we used for reference customization:

"For this, the reference genome sequence GRCm38 was customized for each BXD line with BXD-specific genotypes (from GeneNetwork and imputed) using vcf2diploid software (version 0.2.6) with a slight modification to change the software's behaviour with unphased heterozygous variants. Prior to compiling the software according to the installation instructions, we removed the function that randomizes unphased heterozygous variants (to determine whether there are included in the paternal or maternal genome) and the call to this function (see Table 1). It is, however, entirely possible to use the software without these modifications." (lines 180-187)

7) The study describes work on inbred mice however what would your recommendations be for human as an outbred example? Or is your approach only be suitable for inbred organisms?

Our approach was greatly inspired by methods developed for human research (e.g. the vcf2diploid tool) where variants are individually and often more completely characterized (heterozygous mutation, CNVs, phasing of heterozygous variants) than in mice. The vcf2diploid tool can be used to generate an individualized reference. In the case where the individual variants are unknown, but are known for the parents of the individual, the AlleleSeq pipeline (of which vcf2diploid is part of) offers the possibility for reads to be mapped on the parental alleles it may originate from, while making sure that each read is counted only once. The method consists of the following steps:

1) Generate a reference for each parent based their variants.
2) Map the individual reads on both references separately.
3) Compare for each read if it maps better (higher mapping score of the aligner) on one reference than the other.
    a. If the scores are different between references, keep for this read only the alignment to the reference with the highest score.
    b. If the scores are identical between references, pick randomly the alignment to one reference for this read.

Minor comments:

1) L129: "Assembly refers to genomic sequences assembled from DNA reads to form

chromosomes whereas reference simply refers to the sequences the reads are compared to during read mapping" -> This needs to be clearer.

This sentence was removed in the process of clarification of the entire method section.

2) L325 "we modified the B6 reference assembly using SNPs and indels specific to the D2 strain from dbSNP. We mapped parental and F1 samples on these two assemblies with exact matches." -> what 2 assemblies, mm10 and modified?

We modified the text as follows: "We mapped parental and F1 samples with exact matches on both the mm10 assembly and the mm10 assembly modified for D2."

3) L332: "D2 samples gained between 0.0% and 3.4%" -> You don't gain 0% and also why where there no gains in this instance?

More reads were uniquely mapped in all D2 samples on the D2 assembly compared to GRCm38 (i.e. 'gain'), however the increase in the percentage of uniquely mapped reads was very small in some samples, with the lowest being 0.03% which was rounded to 0.0%. For clarity, we put the unrounded value in the results as following: "D2 samples gained between 0.03% and 3.4%" (lines 347-348).

4) In Figure 5 you aim to maximise eQTLs. Can you explain why more eQTLs are indicative of better performance?

eQTL are associations between genetic variants and gene expression. Such covariation may exist because the genetic variant influences the expression of the gene. Obtaining more eQTLs is indicative of a more genetically structured gene expression versus random noise. This metric is also used elsewhere (Munger et al., 2014; Rubinacci et al., 2021). We have explained the rational for using this metric in the manuscript in the Results section: "The ratio of significant eQTLs to expressed genes (Figure 5) was used as elsewhere (Munger et al., 2014; Rubinacci et al., 2021). A higher ratio indicates a higher proportion of genetically structured gene expression versus random variations in gene expression." (lines 424-427).

5) L434 "However, even though it has been shown to importantly impact genetic specificity (Yuan et al., 2015)" -> What exactly is meant by "genetic specificity"? Is it reads uniquely mapped? Regardless, it need to be clearly defined

These authors showed that the difference in uniquely mapped reads between a classical assembly ("universal") and a customized reference becomes larger when 0 mismatches are allowed compared to 1 and 2 mismatches. Moreover, this difference is also larger for reads with alternative alleles than reference alleles.

We rephrased this statement as follows: "However, the choice of the number of mismatches allowed is rarely given, even though it has been shown in humans that reducing the number of mismatches allowed increased the difference in uniquely mapped reads when using a general versus an individual-specific reference (Yuan et al., 2015)." (lines 464-467).

6) L449: "Indeed, each BXD sample has both B6 and D2 alleles, so the samples compared for

each gene are not split the same way, depending on alleles at this locus." -> What does "depending on alleles at this locus" mean?

Two groups are needed to perform differential gene expression (e.g. control vs. treatment; strain A vs. strain B; etc). In our case, the gene expression comparison is between the two parental alleles which segregate among the BXD lines. To assess differential gene expression in this panel, samples have to be split differently for each gene according to the alleles (B6 vs. D2) of nearby markers.

This has now been rephrased as: "We used the fraction of eQTLs as evaluation measure rather than DE, because we compared B6 versus D2 alleles in a genetic population and not two fixed groups. Indeed, each BXD sample will be considered to be B6 at some loci and D2 at others." (lines 476-479)

I found a few typos / poorly worded sentences throughout. A few are highlighted below although I would suggest a thorough proof read.

We have improved the phrasing throughout the manuscript.

Example typos and sentence structures:

L140: "sequences are not containing alternative haplotypes" -> do not contain

Corrected.

L166: "only one BXD lines" -> line

Corrected.

L384: "However, we assumed that globally B6 alleles are equally expressed than D2 alleles. Moreover, since our samples are inbred lines, the heterozygous sites are greatly rarer" -> Two errors here

We rewrote the sentences as follows: "However, we assumed that globally genes with B6 or D2 alleles are equally expressed. Moreover, since our samples are from inbred lines, heterozygous sites are rarer than in other populations, so we compared homozygous alleles of genetically different samples, rather than heterozygous alleles from one sample." (lines 410-414).

L427: "However, this does not guaranty" -> guarantee

Corrected.

L428: "due to e.g. redundancy in the genome" -> not sure what this means

We rewrote the sentences as follows: "However, this does not guarantee the correctness of mapping of uniquely mapping reads. Importantly, some reads are expected to be correctly classified as multi-mappers because some regions in the genome are identical or very similar (e.g. repeat elements)." (lines 457-459). We also removed an occurrence of the expression "redundancy within the genome", now: "The mapping needs to account for amplification and sequencing errors, and for repeated sequences within the genome." (lines 37-38).

The mapping needs to account for amplification and sequencing errors, and for redundancy within the genome.

Reviewer #2: This manuscript describes the importance of the reference genome chosen when aligning short read data for subsequent analysis in RNAseq experiments. The insight from the recombinant inbred strains is particularly interesting. The authors present information of how this may skew findings, especially the results of the differential mapping analysis (Fig 2D and 3B). However, some basic analyses need to be performed to increase the credibility of the results. Especially surrounding the mappability analyses. Also, the manuscript has significant problems in execution, namely the methods. Some of the points raised in the discussion could actually be quickly tested by the authors with existing data.

We thank reviewer 2 for their comments and for highlighting the importance of our work.

Methods:

The methods are not publication-quality and are confusing. These need to be re-written for improved understandability and credibility.

We have rewritten the Methods and added more non-technical explanations and we hope they are now less confusing.

Results:

At line 287, the authors note surprising deviations from their expectations of mappability of reads from B6, D2 and BXD lines. One possible interpretation that is not addressed is the possible mixups with individual mice and/or tubes when the original tissue samples were collected. Also, we have seen cross-contamination of samples introduced at sequencing facilities, even including the identification of reads from different mammalian species. The transcriptomic data is from pooled samples from many individual mice and an accidental inclusion of one or mice from the wrong strain into a pooled sample would go some way to explain the results the authors obtained. These sample mix-ups a very common, even in the largest and most automated genome centres. It would be possible to search for reads that contain genotypes that are specific to either B6 or D2 and count the relative presence of these in each sample pool. The presence or absence of contaminating reads (potentially introduced even from the sequencing step) would be instructive to the following results.

We already addressed concerns of cross-contamination for this dataset in an earlier publication (Jan et al. 2019) and have now restated this in the current Methods (lines 118-120). Checking for cross-species contamination is part of our sequencing facility quality control and has been performed for each sample. As to a possible cross-strain contamination samples are pools of a maximum of 3 mice (see Suppl. Table 1). Accidental inclusion of one mouse from a different strain would therefore have an important effect that we would have caught when we checked the consistency between GeneNetwork genotypes and genotypes derived from calling variants on our RNA-seq data (Jan et al., 2019). We copy here the Figure 4 of that publication:

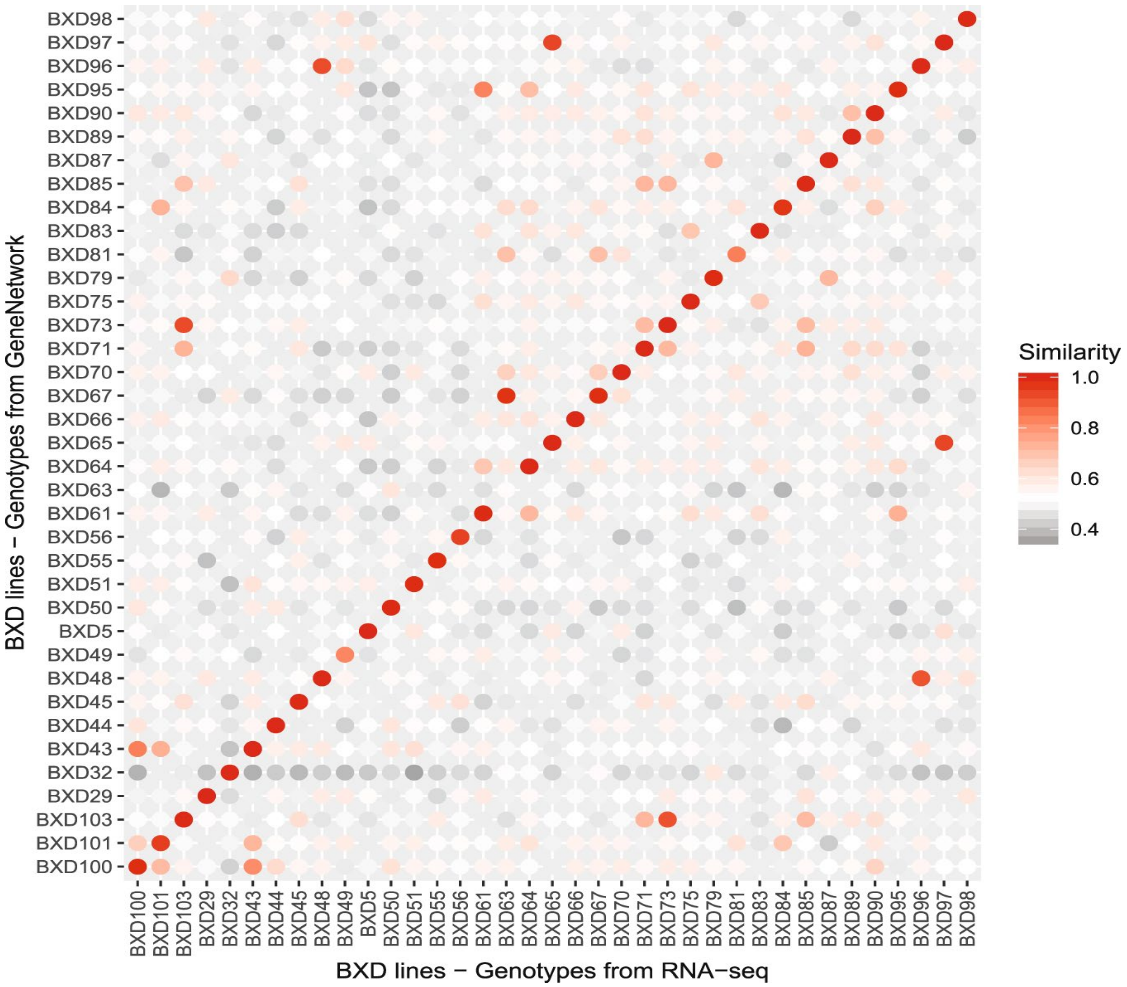

The figure depicts a similarity matrix [in %] between RNA-seq variant calling and GeneNetwork genotypes. A similarity of 1 indicates that all common genotypes are similar. We here compared only genotypes that were labelled as 'B' or 'D' and excluded unknown ('U') or heterozygous ('H') genotypes.

This analysis revealed that all mice labelled BXD63 by the provider were in fact BXD67. We excluded data obtained in these BXD63-labelled mice from all analyses. It can also be seen on the graph that three pairs of lines have strong genetic similarity: BXD48 and BXD96, BXD65 and BXD97, BXD73 and BXD103 (red dots off diagonals). These are known closely related sub-strains that have thus been renamed by Jackson laboratory: BXD96 was redesignated as BXD48a, BXD97 was redesignated as BXD65a, and finally BXD103 was redesignated as BXD73b.

On line 290 the authors also describe mappability differences between the cortex and liver samples in the same lines. That mappability should vary between tissues from the same organism points to a technical difference in the preparation of the samples. The reasons for the differences may be trivial, but the authors need to address this further. For example, are differences in sequencing depth or increased sequencing error important for the mappability differences that are a main focus of the manuscript?

We addressed this further with the information that we have: "We also noticed that mappability differed between the liver and the cortex both in amplitude and in variance. This difference might relate to differences in the preparation of the two tissues for reasons inherent to the tissues, but all the samples

passed the quality tests (Diessler et al., 2018). The liver samples had on average more raw reads than the cortex samples, but the sequencing depth did not seem to explain differences in mappability." (lines 300-304).

The mappability of reads seems to have been assessed in two ways, i) exact matches, and ii) up to ten mismatches. Both might be problematic: Generally, the rate of short-read sequencing error is ~1 nucleotide per 100, in the 100bp reads they have obtained, most of them will have a sequencing error. So, requiring exact matches will be too strict for many otherwise-mappable reads. However, allowing 10 mismatches will not resolve reads that could be mapped to multiple members of gene families. Given the importance of this to the following analysis, some optimisation of the allowed mismatches in alignment is essential. As would be inclusion of comparison with a different aligner. The "Mapping parameters evaluation" section is interesting, but does not address the need to optimise the number of allowed mismatches.

We fully agree with the importance of the number of mismatches allowed. We therefore tested more than just 0 and 10 mismatches, and optimized this parameter as well as others that were important and evaluated their influence on detecting local eQTLs (see Figure 5). The choice of the aligner it-self appears not to be of great importance (see Sha et al. 2015, which we cited on lines 468-473 and our own comparison between the STAR and Kallisto mappers in Figure S1).

Discussion:

On line 438 the authors note the need for more investigation of tissue effects and point to existing data from GTex. Given the relevance of this to the current manuscript, potentially the authors might judiciously choose a small number of example datasets from GTex that include cortex/liver comparisons – and investigate these for tissue effects themselves and include this in the manuscript.

The tissue difference in mappability we observed was an accidental finding and not the main focus of our manuscript. The mention of GTEx is mainly due to the fact that is the largest RNA profiling experiment in human. The ability of our group to access dbGAP control data is beyond our current interest and manpower. We believe by mentioning the GTEx some readers interested in human-mouse comparison we will potentially perform these analyses.

On line 479 of the discussion, the point is made about the genetic drift of inbred lines (10 to 30 germline mutations per generation) and the relevance this has to mappability. Which is true, yet de novo variation accumulated since the production of a reference sequence seems a red herring. The variation mentioned is unlikely to be problematic for read-mapping so soon after the reference genomes have been sequenced. Even after a decade and assuming a very high 10 generations per year, this is only 10x10x30 = 3000 de novo variants distributed among 3 gigabases of genome. This may very likely cause phenotypic divergence. Yet, for alignment of RNAseq data, this will be only around 30 coding de novo variants, hence only a little more than one 1 in 1000 genes are likely to harbour a variant that has occurred since the production of the reference sequence for the same strain.

Thank you for your estimation of coding *de novo* mutations in mouse. Even though it would only affect 15 of the 15'000 genes in a given mouse strain, we nevertheless thought it is a factor worth considering for an optimal reference. To avoid readers to think we imply a huge effect, we removed the following

sentence: “It is likely that B6 samples used to build the GRC assembly and the B6 samples used currently present some genetic drift.”.

References from the answers to reviewers’ comments

Bray, N. L., Pimentel, H., Melsted, P., & Pachter, L. (2016). Near-optimal probabilistic RNA-seq quantification. Nature Biotechnology, 34(5), 525–527. https://doi.org/10.1038/nbt.3519

Dobin, A., Davis, C. A., Schlesinger, F., Drenkow, J., Zaleski, C., Jha, S., Batut, P., Chaisson, M., & Gingeras, T. R. (2013). STAR: Ultrafast universal RNA-seq aligner. Bioinformatics, 29(1), 15–21. https://doi.org/10.1093/bioinformatics/bts635

Jan, M., Gobet, N., Diessler, S., Franken, P., & Xenarios, I. (2019). A multi-omics digital research object for the genetics of sleep regulation. Scientific Data, 6(1), 1–15. https://doi.org/10.1038/s41597-019-0171-x

Munger, S. C., Raghupathy, N., Choi, K., Simons, A. K., Gatti, D. M., Hinerfeld, D. A., Svenson, K. L., Keller, M. P., Attie, A. D., Hibbs, M. A., Graber, J. H., Chesler, E. J., & Churchill, G. A. (2014). RNA-Seq Alignment to Individualized Genomes Improves Transcript Abundance Estimates in Multiparent Populations. Genetics, 198(1), 59–73. https://doi.org/10.1534/genetics.114.165886

Rubinacci, S., Ribeiro, D. M., Hofmeister, R. J., & Delaneau, O. (2021). Efficient phasing and imputation of low-coverage sequencing data using large reference panels. Nature Genetics, 53(1), 120–126. https://doi.org/10.1038/s41588-020-00756-0

Sha, Y., Phan, J. H., & Wang, M. D. (2015). Effect of low-expression gene filtering on detection of differentially expressed genes in RNA-seq data. Conference Proceedings : ... Annual International Conference of the IEEE Engineering in Medicine and Biology Society. IEEE Engineering in Medicine and Biology Society. Annual Conference, 2015, 6461–6464. https://doi.org/10.1109/EMBC.2015.7319872

Yuan, S., Johnston, H. R., Zhang, G., Li, Y., Hu, Y.-J., & Qin, Z. S. (2015). One Size Doesn’t Fit All - RefEditor: Building Personalized Diploid Reference Genome to Improve Read Mapping and Genotype Calling in Next Generation Sequencing Studies. PLOS Computational Biology, 11(8), e1004448. https://doi.org/10.1371/journal.pcbi.1004448
