# Supplementary material for: Towards mouse genetic-specific RNA-sequencing read mapping
Source: PLoS Comput Biol. 2022 Sep 26;18(9):e1010552. doi: 10.1371/journal.pcbi.1010552 (PMC9536569; doi:10.1371/journal.pcbi.1010552)
Supplement: Attachment — Submitted filename: Revised_manuscript_comments_220713.pdf [file pcbi.1010552.s009.pdf]

Reviewer #2: This manuscript describes the importance of the reference genome chosen when aligning short read data for subsequent analysis in RNAseq experiments. The insight from the recombinant inbred strains is particularly interesting. The authors present information of how this may skew findings, especially the results of the differential mapping analysis (Fig 2D and 3B). However, some basic analyses need to be performed to increase the credibility of the results. Especially surrounding the mappability analyses. Also, the manuscript has significant problems in execution, namely the methods. Some of the points raised in the discussion could actually be quickly tested by the authors with existing data.

We thank reviewer 2 for their comments and for highlighting the importance of our work.

Methods:
The methods are not publication-quality and are confusing. These need to be re-written for improved understandability and credibility.
We have rewritten the Methods and added more non-technical explanations and we hope they are now less confusing.

The revised Methods are now significantly improved.

Results:

At line 287, the authors note surprising deviations from their expectations of mappability of reads from B6, D2 and BXD lines. One possible interpretation that is not addressed is the possible mixups with individual mice and/or tubes when the original tissue samples were collected. Also, we have seen cross-contamination of samples introduced at sequencing facilities, even including the identification of reads from different mammalian species. The transcriptomic data is from pooled samples from many individual mice and an accidental inclusion of one or mice from the wrong strain into a pooled sample would go some way to explain the results the authors obtained. These sample mix-ups a very common, even in the largest and most automated genome centres. It would be possible to search for reads that contain genotypes that are specific to either B6 or D2 and count the relative presence of these in each sample pool. The presence or absence of contaminating reads (potentially introduced even from the sequencing step) would be instructive to the following results.

We already addressed concerns of cross-contamination for this dataset in an earlier publication (Jan et al. 2019) and have now restated this in the current Methods (lines 118-120). Checking for cross-species contamination is part of our sequencing facility quality control and has been performed for each sample. As to a possible cross-strain contamination samples are pools of a maximum of 3 mice (see Suppl. Table 1). Accidental inclusion of one mouse from a different strain would therefore have an important effect that we would have caught when we checked the consistency between GeneNetwork genotypes and genotypes derived from calling variants on our RNA-seq data (Jan et al., 2019). We copy here the Figure 4 of that publication:

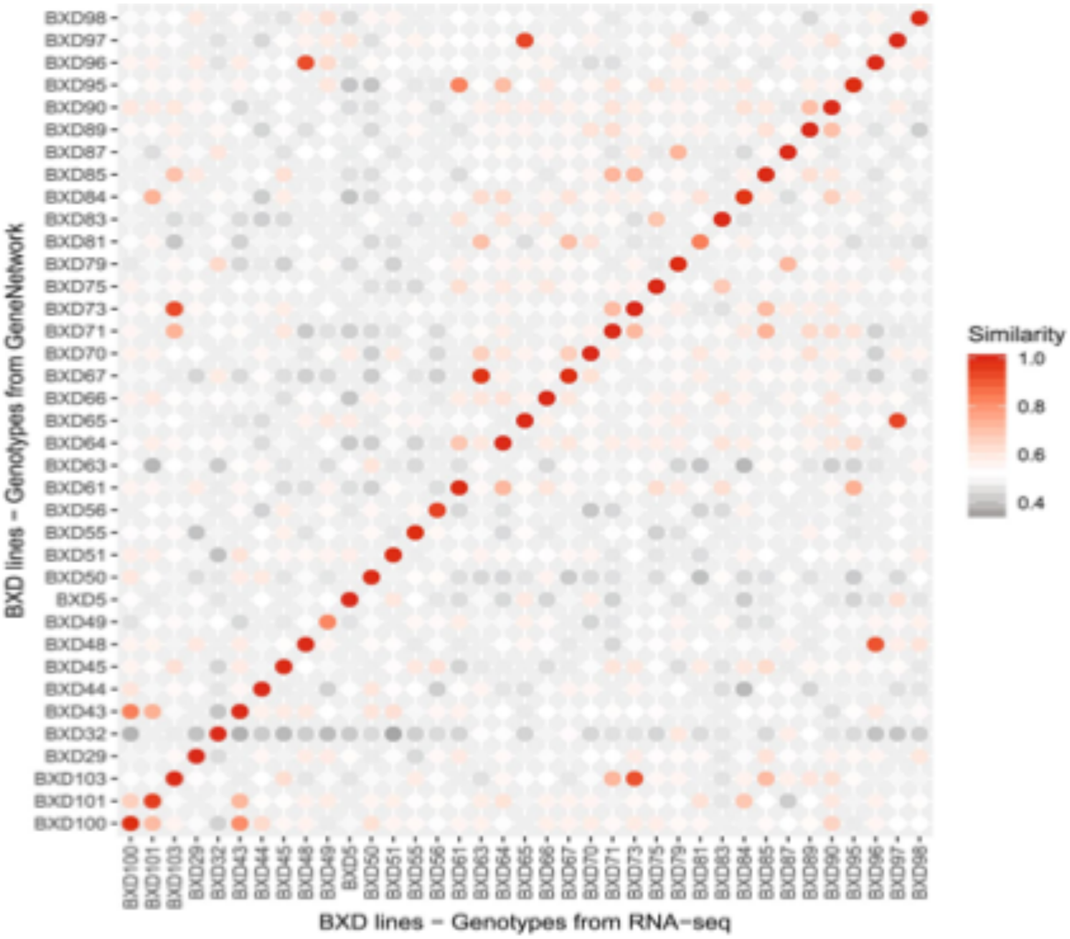

The figure depicts a similarity matrix [in %] between RNA-seq variant calling and GeneNetwork genotypes. A similarity of 1 indicates that all common genotypes are similar. We here compared only genotypes that were labelled as 'B' or 'D' and excluded unknown ('U') or heterozygous ('H') genotypes.

This analysis revealed that all mice labelled BXD63 by the provider were in fact BXD67. We excluded data obtained in these BXD63-labelled mice from all analyses. It can also be seen on the graph that three pairs of lines have strong genetic similarity: BXD48 and BXD96, BXD65 and BXD97, BXD73 and

BXD103 (red dots off diagonals). These are known closely related sub-strains that have thus been renamed by Jackson laboratory: BXD96 was redesignated as BXD48a, BXD97 was redesignated as BXD65a, and finally BXD103 was redesignated as BXD73b.

This is interesting and a convincing demonstration that sample swaps are not the cause of this surprising results. This information is not included prominently in the current manuscript. The present revised manuscript is still not lucid without first being familiar with this previous published work. Potentially a sentence or two indicating in the manuscript what they lay out here would be useful to readers.

On line 290 the authors also describe mappability differences between the cortex and liver samples in the same lines. That mappability should vary between tissues from the same organism points to a technical difference in the preparation of the samples. The reasons for the differences may be trivial, but the authors need to address this further. For example, are differences in sequencing depth or increased sequencing error important for the mappability differences that are a main focus of the manuscript?

We addressed this further with the information that we have: "We also noticed that mappability differed between the liver and the cortex both in amplitude and in variance. This difference might relate to differences in the preparation of the two tissues for reasons inherent to the tissues, but all the samples passed the quality tests (Diessler et al., 2018). The liver samples had on average more raw reads than the cortex samples, but the sequencing depth did not seem to explain differences in mappability." (lines 300-304).

This is an important addition to the information present in the original manuscript. Can the authors speculate what might be causing the differential mappability between tissue preparations? Are there more mismatches/errors in one sequence dataset than the other?

The mappability of reads seems to have been assessed in two ways, i) exact matches, and ii) up to ten mismatches. Both might be problematic: Generally, the rate of short-read sequencing error is ~1 nucleotide per 100, in the 100bp reads they have obtained, most of them will have a sequencing error. So, requiring exact matches will be too strict for many otherwise-mappable reads. However, allowing 10 mismatches will not resolve reads that could be mapped to multiple members of gene families. Given the importance of this to the following analysis, some optimisation of the allowed mismatches in alignment is essential. As would be inclusion of comparison with a different aligner. The "Mapping parameters evaluation" section is interesting, but does not address the need to optimise the number of allowed mismatches.

We fully agree with the importance of the number of mismatches allowed. We therefore tested more than just 0 and 10 mismatches, and optimized this parameter as well as others that were important and evaluated their influence on detecting local eQTLs (see Figure 5). The choice of the aligner it-self appears not to be of great importance (see Sha et al. 2015, which we cited on lines 468-473 and our own comparison between the STAR and Kallisto mappers in Figure S1).

It still remains unclear how the mismatch thresholds presented in Figure 5 were chosen. For instance, as the allowed mismatches parameter was optimised, how was the improvement measured? Was this the relative counts of reads aligned to a specific gene/transcript? As the allowed mismatches parameter is optimised, many reads may differentially map to other gene copies present – how was this effect measured? Many of the reads are mapped to eQTLs with up to

10 allowed mismatches and for very many genes, allowing a 10% mapping mismatch will allow a read to map to all members of a gene family.

Discussion:

On line 438 the authors note the need for more investigation of tissue effects and point to existing data from GTex. Given the relevance of this to the current manuscript, potentially the authors might judiciously choose a small number of example datasets from GTex that include cortex/liver comparisons – and investigate these for tissue effects themselves and include this in the manuscript.

The tissue difference in mappability we observed was an accidental finding and not the main focus of our manuscript. The mention of GTEx is mainly due to the fact that is the largest RNA profiling experiment in human. The ability of our group to access dbGAP control data is beyond our current interest and manpower. We believe by mentioning the GTEx some readers interested in human-mouse comparison we will potentially perform these analyses.

This is noted, though remains a good way to replicate some of the work done in this manuscript, which would strengthen the results.

On line 479 of the discussion, the point is made about the genetic drift of inbred lines (10 to 30 germline mutations per generation) and the relevance this has to mappability. Which is true, yet de novo variation accumulated since the production of a reference sequence seems a red herring. The variation mentioned is unlikely to be problematic for read-mapping so soon after the reference genomes have been sequenced. Even after a decade and assuming a very high 10 generations per year, this is only 10x10x30 = 3000 de novo variants distributed among 3 gigabases of genome. This may very likely cause phenotypic divergence. Yet, for alignment of RNAseq data, this will be only around 30 coding de novo variants, hence only a little more than one 1 in 1000 genes are likely to harbour a variant that has occurred since the production of the reference sequence for the same strain.

Thank you for your estimation of coding *de novo* mutations in mouse. Even though it would only affect 15 of the 15’000 genes in a given mouse strain, we nevertheless thought it is a factor worth considering for an optimal reference. To avoid readers to think we imply a huge effect, we removed the following

sentence: “It is likely that B6 samples used to build the GRC assembly and the B6 samples used currently present some genetic drift.”.

This is strictly true, but this comment does not point out that it will be of negligible importance.
