# Supplementary material for: Towards mouse genetic-specific RNA-sequencing read mapping
Source: PLoS Comput Biol. 2022 Sep 26;18(9):e1010552. doi: 10.1371/journal.pcbi.1010552 (PMC9536569; doi:10.1371/journal.pcbi.1010552)
Supplement: Attachment — Submitted filename: Revised_manuscript_comments_answers2.pdf [file pcbi.1010552.s010.pdf]

We again thank Reviewer #2 for the constructive feedback. Please see below for our response to the few remaining issues. To have a stand-alone document we kept the comments that went into two rounds of review. Here is a legend to the different fonts used to distinguish the two rounds and the reviewer from our comments:

- Reviewer comments round 1
- Authors responses round 1
- Reviewer comments round 2
- Authors responses round 2

We also took this opportunity to improve the text with a few minor edits. These edits and the changes made in response to the reviewer's comments can be found in the 'redline' copy.

Response to comments from Reviewer 2:

Methods:

The methods are not publication-quality and are confusing. These need to be re-written for improved understandability and credibility.

We have rewritten the Methods and added more non-technical explanations and we hope they are now less confusing.

The revised Methods are now significantly improved.

Thank you for helping the clarification of the methods.

Results:

At line 287, the authors note surprising deviations from their expectations of mappability of reads from B6, D2 and BXD lines. One possible interpretation that is not addressed is the possible mixups with individual mice and/or tubes when the original tissue samples were collected. Also, we have seen cross-contamination of samples introduced at sequencing facilities, even including the identification of reads from different mammalian species. The transcriptomic data is from pooled samples from many individual mice and an accidental inclusion of one or mice from the wrong strain into a pooled sample would go some way to explain the results the authors obtained. These sample mix-ups a very common, even in the largest and most automated genome centres. It would be possible to search for reads that contain genotypes that are specific to either B6 or D2 and count the relative presence of these in each sample pool. The presence or absence of contaminating reads (potentially introduced even from the sequencing step) would be instructive to the following results.

We already addressed concerns of cross-contamination for this dataset in an earlier publication (Jan et al. 2019) and have now restated this in the current Methods (lines 118-120). Checking for cross-species contamination is part of our sequencing facility quality control and has been performed for each sample. As to a possible cross-strain contamination samples are pools of a maximum of 3 mice (see Suppl. Table 1). Accidental inclusion of one mouse from a different strain would therefore have an important effect that we would have caught when we checked the consistency between GeneNetwork genotypes and genotypes derived from calling variants on our RNA-seq data (Jan et al., 2019). We copy here the Figure 4 of that publication:

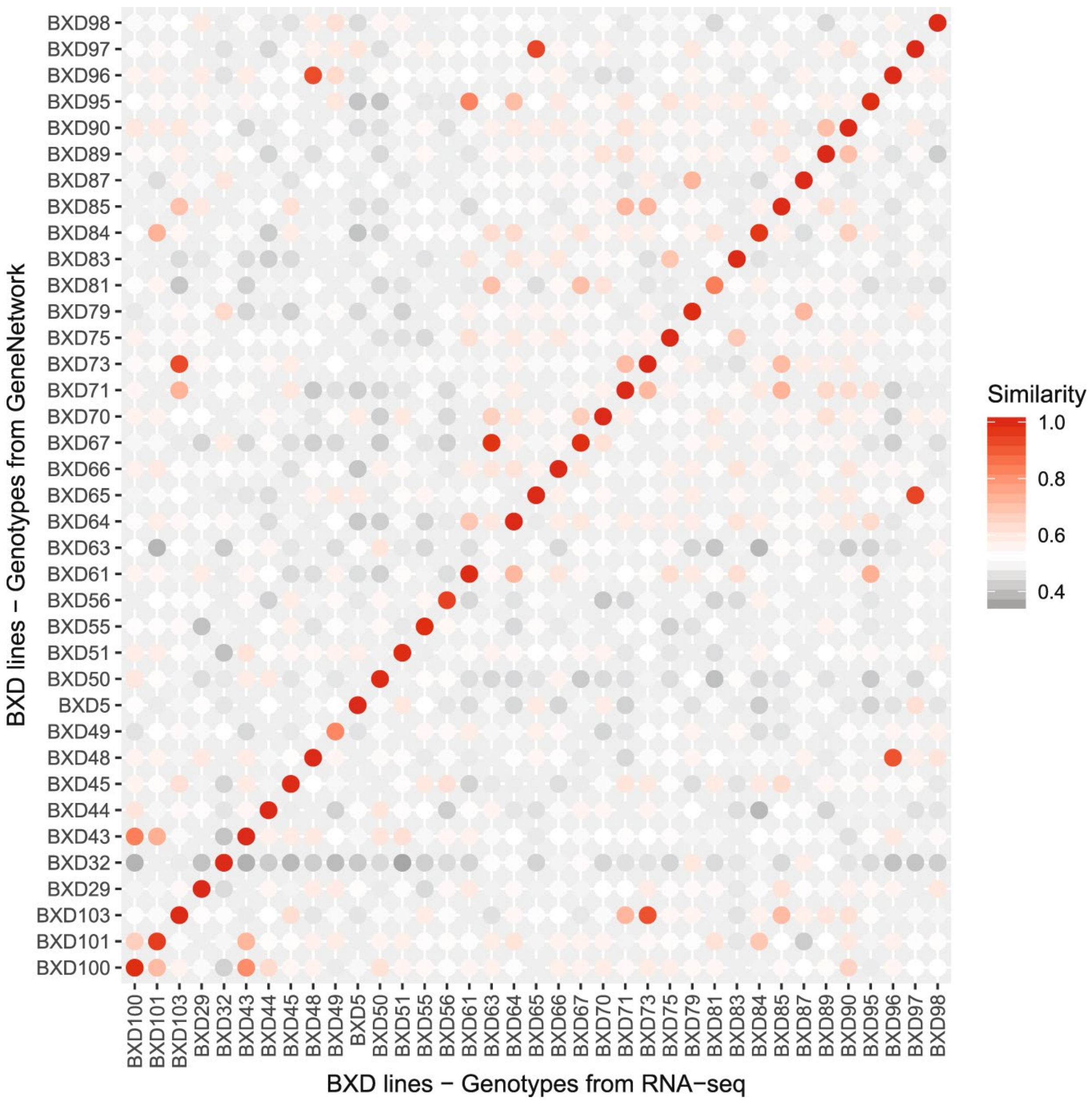

BXD lines − Genotypes from GeneNetwork
BXD98
BXD97
BXD96
BXD95
BXD90
BXD89
BXD87
BXD85
BXD84
BXD83
BXD81
BXD79
BXD75
BXD73
BXD71
BXD70
BXD67
BXD66
BXD65
BXD64
BXD63
BXD61
BXD56
BXD55
BXD51
BXD50
BXD5
BXD49
BXD48
BXD45
BXD44
BXD43
BXD32
BXD29
BXD103
BXD101
BXD100
BXD100 BXD101 BXD103 BXD29 BXD32 BXD43 BXD44 BXD45 BXD48 BXD49 BXD5 BXD50 BXD51 BXD55 BXD56 BXD61 BXD63 BXD64 BXD65 BXD66 BXD67 BXD70 BXD71 BXD73 BXD75 BXD79 BXD81 BXD83 BXD84 BXD85 BXD87 BXD89 BXD90 BXD95 BXD96 BXD97 BXD98
BXD lines − Genotypes from RNA−seq
Similarity
1.0
0.8
0.6
0.4

The figure depicts a similarity matrix [in %] between RNA-seq variant calling and GeneNetwork genotypes. A similarity of 1 indicates that all common genotypes are similar. We here compared only genotypes that were labelled as 'B' or 'D' and excluded unknown ('U') or heterozygous ('H') genotypes.

This analysis revealed that all mice labelled BXD63 by the provider were in fact BXD67. We excluded data obtained in these BXD63-labelled mice from all analyses. It can also be seen on the graph that three pairs of lines have strong genetic similarity: BXD48 and BXD96, BXD65 and BXD97, BXD73 and BXD103 (red dots off diagonals). These are known closely related sub-strains that have thus been renamed by Jackson laboratory: BXD96 was redesignated as BXD48a, BXD97 was redesignated as BXD65a, and finally BXD103 was redesignated as BXD73b.

This is interesting and a convincing demonstration that sample swaps are not the cause of this surprising results. This information is not included prominently in the current manuscript. The present revised manuscript is still not lucid without first being familiar with this previous published work. Potentially a sentence or two indicating in the manuscript what they lay out here would be useful to readers.

We added a sentence in the methods to explain that we also checked for sample swaps and further guide interested readers to the previous work. The quality control and check for mix-up samples sentences are now the following: "All samples passed quality control (Diessler et al., 2018). The eventuality of a mix-up of samples between strains, was tested previously by comparing the similarity between RNA-seq variant calling and GeneNetwork genotypes (see Figure 4 in Jan et al., 2019)." (lines 138-141). To clarify to readers not familiar with our previous published work that the effect observed is not due to sample swaps, we also added the following sentence in the discussion: "In this context it can be noted that we can exclude mix-up of samples as a possible cause of D2 bias in assembly mapping, because it affected all samples: BXD lines, B6 and D2, and because our previous analysis confirmed that this had likely not occurred (Jan et al., 2019)." (lines 498-501).

On line 290 the authors also describe mappability differences between the cortex and liver samples in the same lines. That mappability should vary between tissues from the same organism points to a technical difference in the preparation of the samples. The reasons for the differences may be trivial, but the authors need to address this further. For example, are differences in sequencing depth or increased sequencing error important for the mappability differences that are a main focus of the manuscript?

We addressed this further with the information that we have: "We also noticed that mappability differed between the liver and the cortex both in amplitude and in variance. This difference might relate to differences in the preparation of the two tissues for reasons inherent to the tissues, but all the samples passed the quality tests (Diessler et al., 2018). The liver samples had on average more raw reads than the cortex samples, but the sequencing depth did not seem to explain differences in mappability." (lines 300-304).

This is an important addition to the information present in the original manuscript. Can the authors speculate what might be causing the differential mappability between tissue preparations? Are there more mismatches/errors in one sequence dataset than the other?

Although the RNA of both tissues was of good quality, tissue difference in mappability might nevertheless be due to minor differences in the quality of the extracted RNA, possibly related to differences in tissue composition. A different mappability dependent on the genes expressed is also possible. We have now added in the manuscript these speculations: "It may be that there were more PCR artefacts in the liver reads, so they were either unmapped or multi-mapped which means there were less uniquely mapped reads than in the cortex. Another possibility is that the liver expresses more genes that have regions that are not unique, so more reads are multi-mapped." (lines 318-322). We do not know the number of errors in the sequences as we do not know the 'ground truth' about these sequences. With STAR default parameters (up to 10 mismatches allowed), the mismatch rate per base did not differ between liver and cortex samples.

The mappability of reads seems to have been assessed in two ways, i) exact matches, and ii) up to ten mismatches. Both might be problematic: Generally, the rate of short-read sequencing error is ~1 nucleotide per 100, in the 100bp reads they have obtained, most of them will have a sequencing error. So, requiring exact matches will be too strict for many otherwise-mappable reads. However, allowing 10 mismatches will not resolve reads that could be mapped to multiple members of gene families. Given the importance of this to the following analysis, some optimisation of the allowed mismatches in alignment is essential. As would be inclusion of comparison with a different aligner. The "Mapping parameters evaluation" section is interesting, but does not address the need to optimise the number of allowed mismatches.

We fully agree with the importance of the number of mismatches allowed. We therefore tested more than just 0 and 10 mismatches, and optimized this parameter as well as others that were important and evaluated their influence on detecting local eQTLs (see Figure 5). The choice of the aligner it-self appears not to be of great importance (see Sha et al. 2015, which we cited on lines 468-473 and our own comparison between the STAR and Kallisto mappers in Figure S1).

It still remains unclear how the mismatch thresholds presented in Figure 5 were chosen. For instance, as the allowed mismatches parameter was optimised, how was the improvement measured? Was this the relative counts of reads aligned to a specific gene/transcript? As the allowed mismatches parameter is optimised, many reads may differentially map to other gene copies present – how was this effect measured? Many of the reads are mapped to eQTLs with up to 10 allowed mismatches and for very many genes, allowing a 10% mapping mismatch will allow a read to map to all members of a gene family.

1) The different values for the number of mismatches allowed (0, 1, 2, 3, and 10) were chosen to have a range of stringency (0 being the most stringent, 10 the least), with 10 being the default STAR value for reads of 100 bp.

2) The percentage of expressed genes that have a significant local eQTL is how we measured the improvement for the optimization of the four parameters, including the number of mismatches allowed (see Figure 5). We added a sentence to specify this in the methods: "The percentage of expressed genes that have a significant local eQTL serves to measures the improvement between the different values used for the mapping parameters tested in the evaluation." (lines 267-269).

3) The reads were not aligned to a specific gene/transcript, we aligned reads to the entire genome.

4) We counted the number of uniquely mapped reads on each gene for each different value of allowed mismatches tested. Reads mapping equally on two or more copies of a gene were discarded (not included in the counting for gene expression with this specific value of allowed mismatches). We modified a sentence in the methods to specify HTseq only counts uniquely mapped reads, it now appears as: "To count the uniquely mapped reads per gene after STAR, HTseq (version 0.6.1p1) was used with samtools (version 1.9) to convert alignments from bam to sam format." (lines 241-242).

5) First, it should be noted that even when we set the STAR alignment to allow up to 10 mismatches, it will still look for an exact match if it can find one, and alignments with 10 mismatches are considered inferior (i.e., lower alignment score). Therefore, reads that are an exact match to a unique locus in the reference will always be uniquely mapped independent of how many mismatches were allowed. To come back to your example of a read coming from a gene that belongs to family of closely related genes. Either this read is specific enough to map to its gene of origin and thus has a better alignment to one locus in the genome, or it is not specific enough to be sure that the gene of origin was expressed or another member of the gene family. In this second case, the read can either map equally to multiple members of the gene family (multi-mapper read) so it will be not included to quantify gene expression, or it will randomly map better to one of the other members and add to noise, or the read will be unmapped, which is however less likely with 10 allowed mismatches and multiple family members. To summarize, allowing more mismatches will recover more reads that have sequencing errors or unknown variants to map to the gene they originate from. However, allowing too many mismatches risks that reads that are not sufficiently specific will be recovered and randomly mapped at some locus thereby adding to the noise.

We have added a sentence in the discussion to clarify the role of mismatches in the alignment algorithm: “Mismatches have the task to compensate for sequencing errors or small unknown variants to give some flexibility in case an exact match is not found.” (lines 477-479).

Discussion:

On line 438 the authors note the need for more investigation of tissue effects and point to existing data from GITEX. Given the relevance of this to the current manuscript, potentially the authors might judiciously choose a small number of example datasets from GTex that include cortex/liver comparisons – and investigate these for tissue effects themselves and include this in the manuscript.

The tissue difference in mappability we observed was an accidental finding and not the main focus of our manuscript. The mention of GTEx is mainly due to the fact that is the largest RNA profiling experiment in human. The ability of our group to access dbGAP control data is beyond our current interest and manpower. We believe by mentioning the GTEx some readers interested in human-mouse comparison we will potentially perform these analyses.

This is noted, though remains a good way to replicate some of the work done in this manuscript, which would strengthen the results.

We agree and we have rephrased the sentences in the discussion as follows: “One of these observations concerned the unanticipated tissue differences in mappability. We were unable to identify supportive literature for this phenomenon and think it merits a more thorough analysis addressing such tissue effects (e.g. using GTEx).” (lines 465-467).

On line 479 of the discussion, the point is made about the genetic drift of inbred lines (10 to 30 germline mutations per generation) and the relevance this has to mappability. Which is true, yet de novo variation accumulated since the production of a reference sequence seems a red herring. The variation mentioned is unlikely to be problematic for read-mapping so soon after the reference genomes have been sequenced. Even after a decade and assuming a very high 10 generations per year, this is only 10x10x30 = 3000 de novo variants distributed among 3 gigabases of genome. This may very likely cause phenotypic divergence. Yet, for alignment of RNAseq data, this will be only around 30 coding de novo variants, hence only a little more than one 1 in 1000 genes are likely to harbour a variant that has occurred since the production of the reference sequence for the same strain.

Thank you for your estimation of coding *de novo* mutations in mouse. Even though it would only affect 15 of the 15’000 genes in a given mouse strain, we nevertheless thought it is a factor worth considering for an optimal reference. To avoid readers to think we imply a huge effect, we removed the following sentence: “It is likely that B6 samples used to build the GRC assembly and the B6 samples used currently present some genetic drift.”.

This is strictly true, but this comment does not point out that it will be of negligible importance.

Although the contribution of spontaneous mutations on B6 on the overall mappability are likely to be negligible we simply wanted to indicate that these mutations were not integrated in our approach. We clarified the paragraph in the discussion in two ways:

- We removed the “non-neglectable rate of mutations” and just keep the factual estimation: “germline mutations are estimated to be between 10 and 30 per generation (Casellas & Medrano, 2008; Reardon, 2017)” (lines 527-528).
- We highlighted that the spontaneous mutations effect will likely affect only a few genes in the sentence that now appears as follows: “We made an effort to improve the D2 parts of reference to map the BXD samples, however the B6 strain itself is also susceptible to mutations, as confirmed by the occurrence of many B6 substrains, even if it affects only a few genes.” (lines 539-542).

We think this addresses this caveat of our approach without giving it too much importance.
